# Atrial Ectopy Increases Asynchronous Activation of the Endo- and Epicardium at the Right Atrium

**DOI:** 10.3390/jcm9020558

**Published:** 2020-02-18

**Authors:** Lisette J.M.E. van der Does, Rohit K. Kharbanda, Christophe P. Teuwen, Paul Knops, Charles Kik, Ad J.J.C. Bogers, Natasja M.S. de Groot

**Affiliations:** 1 Department of Cardiology, Erasmus Medical Center Rotterdam, 3015 GD Rotterdam, The Netherlandsr.kharbanda@erasmusmc.nl (R.K.K.); c.teuwen@erasmusmc.nl (C.P.T.); p.knops@erasmusmc.nl (P.K.); 2 Department of Cardiothoracic Surgery, Erasmus Medical Center Rotterdam, 3015 GD Rotterdam, The Netherlands; c.kik@erasmusmc.nl (C.K.);

**Keywords:** atrial tachyarrhythmia, atrial extrasystoles, endo-epicardial asynchrony

## Abstract

The predisposition of atrial extrasystoles (AES) to trigger cardiac tachyarrhythmia may arise from intramural conduction disorders causing endo-epicardial asynchrony (EEA). This study aimed to determine whether spontaneous AES disturb endo-epicardial conduction. Simultaneous endo-epicardial mapping of the right atrium was performed in patients during cardiac surgery with two 128-electrode arrays. Sixty spontaneous AES were observed in 23 patients and were analyzed for incidence of conduction delay, conduction block and amount of EEA compared to the previous sinus rhythm beat. Both conduction delay and block occurred more often in AES compared to sinus rhythm. The difference in lines of conduction block between the epicardium and endocardium increased in AES causing a greater imbalance of conduction disorders between the layers. The incidence of EEA with differences ≥10 ms increased significantly in AES. AES caused delays between the epicardium and endocardium up to 130 ms and EEA to increase for up to half (47%) of the mapping area. Conduction disturbances between the epicardial and endocardial layer giving rise to EEA increase during AES. Asynchronous activation of the atrial layers increases during AES which may be a mechanism for triggering cardiac tachyarrhythmia under the right conditions but EEA cannot be recognized by current mapping tools.

## 1. Introduction

Atrial extrasystoles (AES) are a common finding in the general population and usually the occurrence of AES is benign. However, frequent AES have been associated with increased risk of developing atrial fibrillation and paroxysmal episodes of atrial fibrillation are in more than 90% of the cases preceded by an AES [1,2]. The majority of AES triggering atrial fibrillation originate from the left atrium, most frequently the pulmonary veins [3]. Recently, high-resolution epicardial mapping demonstrated that atrial conduction disorders increase during spontaneous AES, which are caused by anisotropy due to their ectopic origin and/or refractoriness of myocardium due to their prematurity [4]. Slowing of conduction can in turn facilitate re-entry and initiate arrhythmia such as atrial fibrillation.

Conduction disorders are not limited to the 2-dimensions of the superficial layers of the atrial wall. During both atrial fibrillation and sinus rhythm conduction between epicardial and endocardial myocardium can be dissociated causing asynchronous activation of the epicardial and endocardial atrial wall [5,6]. Endo-epicardial asynchrony (EEA) and the ensuing breakthrough waves caused by endo-to-epicardial conduction or vice versa have been proposed as a possible mechanism sustaining atrial fibrillation [5]. Based on these previous findings, we hypothesized that during AES endo-epicardial conduction will become more disturbed than during SR and that EEA could be a potential mechanism triggering arrhythmia. Therefore, we investigated the difference of EEA in sinus rhythm beats and spontaneous AES by performing simultaneous endo-epicardial mapping in patients undergoing cardiac surgery.

## 2. Experimental Section

### 2.1. Study Population

Patients participating in the Epic End study (NTR5370), an endo-epicardial mapping study in adult patients undergoing open-heart surgery, were included. This study was approved by the local ethics committee (MEC 2015-373) and all patients gave informed consent prior to inclusion. Twenty-three patients demonstrating AES during mapping were included of whom 17 (74%) underwent surgery for coronary artery disease and 11 (48%) had valvular heart disease. Nine patients (39%) had a history of atrial fibrillation. Clinical characteristics are presented in Supplemental Table S1.

### 2.2. Endo-Epicardial Mapping Procedure

**Figure 1:**
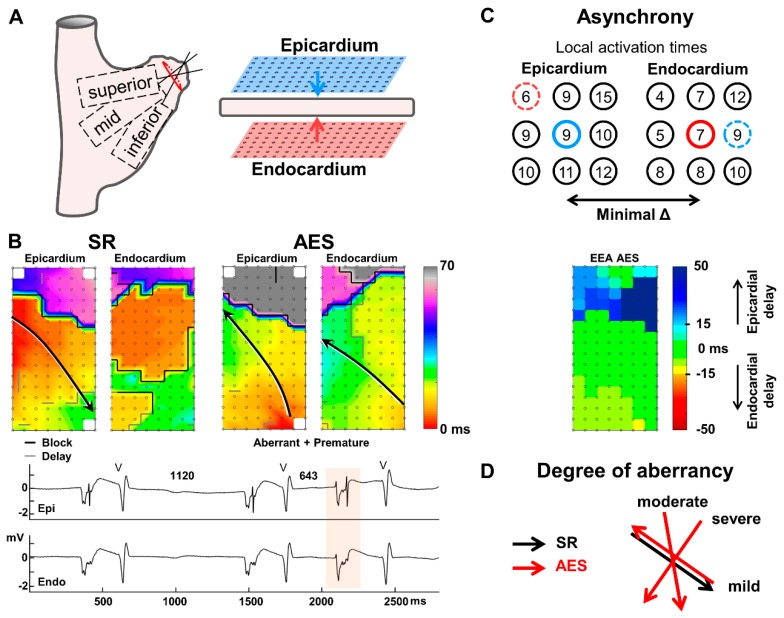
Endo-epicardial mapping and data analysis. (**A**) endo-epicardial mapping was performed with two 128-electrode arrays, one placed on the epicardium and one on the endocardium. Recording locations included (1) the superior right atrium (RA), (2) the mid RA and (3) the inferior RA. (**B**) endo-epicardial activation maps of a sinus rhythm (SR) beat and an atrial extrasystole (AES) show the aberrant activation pattern of the AES, at the epicardium there is mild aberrancy (180° shift of the main wavefront trajectory), at the endocardium there is a complex pattern of activation during sinus rhythm without a main trajectory. Conduction block (Δ > 11 ms) or delay (7–11 ms) between two electrodes represents a line of 2mm. The total length of all lines represents the amount of conduction block and delay. Epi- endocardial electrograms demonstrate the AES is premature as well, with a 57% shortening of the cycle length. V = ventricular activation. (**C**) endo-epicardial asynchrony (EEA) is determined by the minimal local activation time difference between the nine opposite electrodes and the index electrode (center). Electrodes at the border of the array only have ≤6 opposite electrodes and are excluded. At the epicardium the minimal difference is 0 ms (blue), in this example, at the endocardium the minimal difference is −1 ms (red). The maximal difference of two opposite electrodes is shown in the EEA map. Positive values represent an epicardial delay in activation and negatives values an endocardial delay in activation. (**D**) degree of aberrancy is categorized based on the rotational shift of the main trajectory of the AES wavefront; mild 180°, moderate 45°/135°, severe 90°.

Endo-epicardial mapping is performed during surgery prior to start of cardiopulmonary bypass and has been previously described in detail [7]. Two arrays containing 128 electrodes (16 × 8) with 2 mm interelectrode spacing were each fixed on a steel spatula and the spatulas were bound together at the end to preserve precise opposite alignment of the two arrays. After arterial cannulation, one array is introduced in the right atrium for endocardial mapping via the incision for venous cannulation. The second array remains on the outside to record from the epicardium. Simultaneous epi- and endocardial unipolar electrogram recordings of the right atrial free wall were obtained for 5–10 s during sinus rhythm from three locations at the right free atrial wall (Figure 1A). In one case, endo-epicardial mapping was performed at the left atrial appendage before excision.

### 2.3. Data Analysis

Electrogram analysis was performed in MATLAB R2016a (The MathWorks Inc, Natick, MA, USA). Electrograms with injury potentials (baseline elevation after the atrial potential larger than the amplitude of the atrial potential) were excluded from analysis [6]. Recording sites with ≥25% missing or excluded electrograms were not included in this study. Local activation times were marked at the steepest negative slope with a minimal slope of 0.05 mV/ms and a signal-to-noise ratio >2 [6]. Local endo-epicardial time differences were determined for each electrode and consisted of the shortest activation time difference between the electrode and the 9 electrodes on the opposite side (exact opposite electrode and its 8 surrounding electrodes; Figure 1C). Border electrodes (<7 opposite local activation times present) were excluded from analysis. Previously, we defined EEA as activation time difference between epicardium and endocardium of ≥15 ms [5]. As atrial wall thickness is very diverse within the atria, we not only analyzed EEA of ≥15 ms (large EEA) but also moderate EEA between 10–15 ms. EEA in AES is expressed as a percentage of the total electrodes during the AES demonstrating EEA (equaling the area of the array with EEA). Conduction disorders in the superficial epicardial and endocardial planes were defined as the incidence of (1) conduction block with interelectrode difference >11 ms and (2) conduction delay with interelectrode difference of 7–11 ms. Conduction disorders are expressed in mm, translating to the total length of all interelectrode lines of conduction delay and block in the activation maps.

### 2.4. Atrial Extrasystoles

AES were defined as beats during sinus rhythm with either a shortening in cycle length ≥25% to the previous sinus-beat and/or an aberrant activation pattern compared to sinus-beats [4]. Prematurity compared to the sinus-beat (the prematurity index) could not be determined for (1) AES that were the first or second beat of the recording or (2) AES following another AES or (3) in case of AES in bigeminy. AES were labelled for their degree of aberrancy from sinus rhythm, for example, shift in direction of the main wavefront trajectory (wave covering the largest area of the mapping area) (Figure 1B,D). Based on our previous study [4] that demonstrated increased conduction disorders for wavefront shifts of 90°, breakthrough and complex patterns during AES the following categories for aberrancy of AES were defined: (1) no aberrancy (only premature AES) or mild to moderate shift of the main wavefront trajectory in AES (0–45° or 135°–180° shift), (2) severe shift of the main wavefront trajectory in AES (90° shift), breakthrough or complex pattern of activation of AES (and sinus rhythm) and (3) breakthrough or complex pattern of activation in sinus rhythm only. When degree of aberrancy differed between epicardium and endocardium, the severest degree of aberrancy of the two determined the aberrancy category.

### 2.5. Statistical Analysis

Normally distributed data are presented as mean ± SD and skewed data are presented as median (interquartile range). AES mapping data consists of clustered measurements (multiple AES recorded within a patient), and therefore the associations of sinus rhythm and AES with conduction delay, conduction block and EEA were evaluated using Generalized Estimated Equations. The skewed distributions of conduction delay, conduction block and EEA could not be transformed to a normal distribution and were therefore converted to a binary distribution by setting cutoff values that represented a possible shift in occurrence between AES and sinus rhythm as observed in the histograms. The number of positive cases required at least 25% of the data otherwise the cutoff value was set at the top quartile. All cutoff values are presented after the p-value. A binary logistic model was used to assess the effect of AES on conduction delay, conduction block and EEA. Correlation structure was chosen based on the Goodness of Fit in the Quasi Likelihood function. In all cases an independent correlation structure was chosen. Statistical analyses were performed with IBM SPSS Statistics version 21 (IBM corp., Armonk, NY, USA).

## 3. Results

### 3.1. Atrial Extrasystole Characteristics

Twenty-three patients demonstrated 60 AES, per patient a median AES of 2 (min–max: 1–7), and most were recorded at the inferior right atrial free wall (31 at the inferior right atrium vs 12 at the middle and 16 at the superior right atrium and 1 at the left atrium). Ten AES were only aberrant and 9 AES were only premature, the remainder was both aberrant and premature or aberrant with unknown prematurity. The prematurity index could not be determined for 29 AES of the 50 (possibly) premature AES, the remaining 21 premature AES had a prematurity of 45% ± 10%. All premature and/or aberrant AES had a mean interval after the previous beat of 560 ± 135ms (min–max: 340–920 ms). Twenty-five AES (42%) had no to moderate aberrancy, 29 AES (48%) had severe/ complex aberrancy and 6 AES (10%) had a complex pattern of activation on one or both sides during SR only. The majority of AES (37/60) were the second to seventh AES recorded in the same patient. Most of these AES likely originated from the same location within the atria as a previous AES in that same patient, because 26 AES had the same degree of aberrancy as one of those previous AES.

### 3.2. Conduction Delay and Block

During sinus rhythm, the endocardium contained more extensive conduction disorders than the epicardium as total length of conduction block lines was longer at the endocardium (*p* = 0.015, ≥20 mm). Total length of lines with conduction delay was similar for both the epicardium and endocardium. AES increased the total length of these lines of conduction block and delay (Table 1). Conduction block increased during AES from 8 (0–48) mm to 20 (6–41) mm (*p* = 0.020, ≥10 mm). Comparable to conduction block, conduction delay increased as well during AES from 24 (12.5–36) mm to 38 (18.5–50) mm (*p* = 0.014, ≥30 mm). At the epicardium, conduction delay increased from 11 (4–19.5) mm to 16 (8–27.5) mm in AES (*p* = 0.030, CD ≥ 10 mm) whereas conduction block did not change (*p* = 0.639, >16 mm) (Supplemental Figure S1A,C). Total length of conduction block (*p* = 0.113, ≥10 mm) rather than conduction delay (*p* = 0.276, ≥24 mm) tended to increase in AES at the endocardium (Supplemental Figure S1B,C). The difference between the epicardial and endocardial layer in the total length of conduction disorders was also analyzed. The endo-epicardial difference in total length of conduction block increased from 2 (0–8) mm in SR to 10 (2–18) mm during AES (*p* = 0.037, Δ ≥ 14 mm). Endo-epicardial difference of total conduction delay did not change during AES (*p* = 0.102, Δ ≥ 14 mm).

### 3.3. Endo-Epicardial Asynchrony

**Figure 2:**
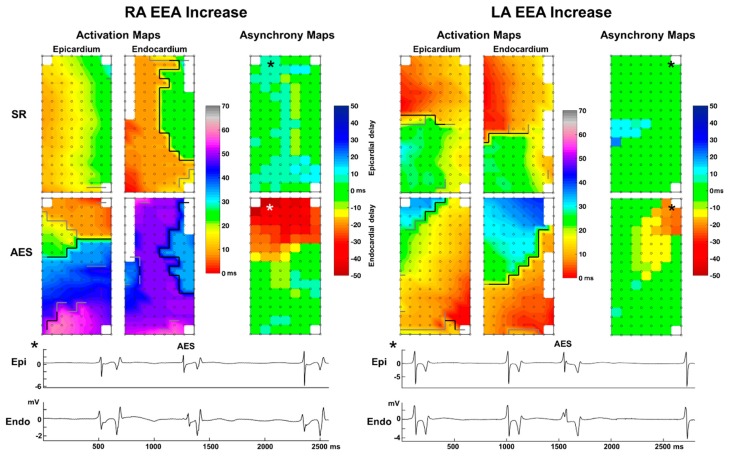
Examples of an increase of endo-epicardial asynchrony (EEA) during atrial extrasystoles (AES) at the right (RA) and left atrium (LA). Color-coded activation maps of epicardial and endocardial local activation times and asynchrony maps are presented for sinus rhythm (SR, top) and atrial extra systole (AES, middle). Grey and black lines represent interelectrode conduction delay and block. Bottom: electrogram examples from the location where EEA increases during the AES (asterisks).

During AES, an increase of EEA occurred almost twice as much as a decrease of EEA. An increase in EEA ≥15 ms occurred in 17 AES (28%) presenting in 8 patients, which included the AES recorded at the left atrium (Figure 2). These AES resulted in EEA with a median delay of 20 ms per AES and 23 ms per patient (min–max: 16–81 ms). The largest endo-epicardial time delay observed at a site during an AES was 130 ms. In 9 AES (15%) recorded from 8 patients EEA decreased compared to sinus rhythm. These AES had EEA during sinus rhythm with a median delay of 16ms per AES and patient (min–max: 15–26 ms). In the remaining 34 AES (57%), EEA did not change.

**Figure 3:**
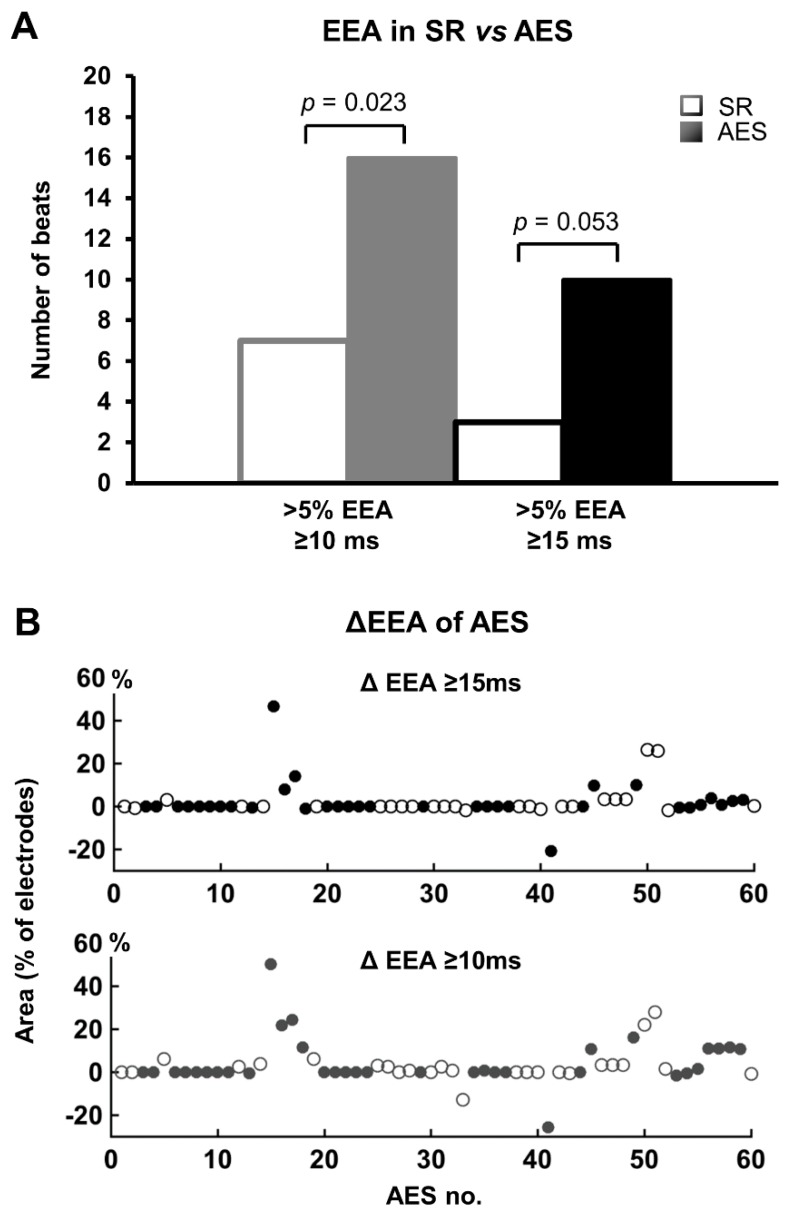
Change in endo-epicardial asynchrony (EEA) during atrial extra systoles (AES). (**A**) frequency histograms of EEA between 10–15 ms and ≥15 ms during sinus rhythm (SR) and AES. Areas of EEA covering >5% of electrodes increase during AES. For EEA ≥10 ms, binary logistic analysis using Generalized Estimated Equations demonstrated a significant association between >5% EEA and AES (*p* = 0.023). (**B**) difference in EEA of AES compared to SR for EEA ≥10 ms and EEA ≥15 ms. Positive values represent an increase of EEA in AES, negative values represent a decrease of EEA in AES. Multiple AES in one patient are clustered and a change from an open (white) to a filled (dark) marker represents a different patient.

Besides EEA of ≥15 ms, also endo-epicardial time differences between 10-15ms were analyzed. Figure 3A shows the number of sinus-beats and AES demonstrating areas of large EEA (≥15 ms) and moderate and large EEA combined (≥10 ms). Incidences for both EEA groups increased during AES for EEA areas >5%. The changes in EEA during each AES for every individual patient are presented in Figure 3B. The top panel includes large EEA (≥15 ms) only and the middle panel includes both moderate and large EEA (≥10 ms). The increase of large EEA during AES almost reached a significant result (*p* = 0.053, EEA area >5%). The most extensive increase of EEA area observed during AES involved 47% of the mapped area. When both moderate and large EEA were included, almost half of all AES (47%) demonstrated an increase in EEA during AES. Incidence of EEA≥10 ms increased during AES (*p* = 0.023, EEA area >5%). 

**Figure 4:**
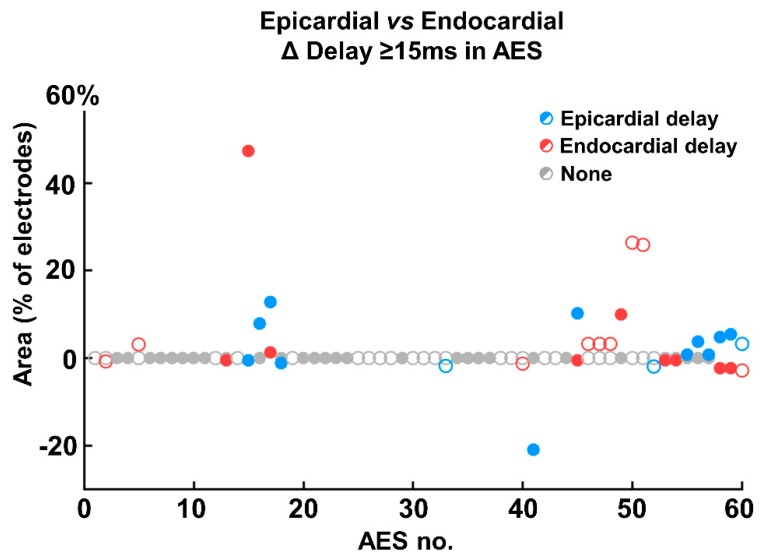
Scatterplot presenting the amount of decrease or increase of endocardial and epicardial delays ≥15 ms per atrial extrasystole (AES) compared to sinus rhythm. Multiple AES in one patient are clustered and a change from an open to a filled marker represents a different patient. For example, in the 8th patient the first AES has a 47% increase in endocardial delays and an 0.5% decrease in epicardial delays. The second AES in this patient demonstrates a 7.8% increase in epicardial delay and no change in endocardial delay

**Figure 5:**
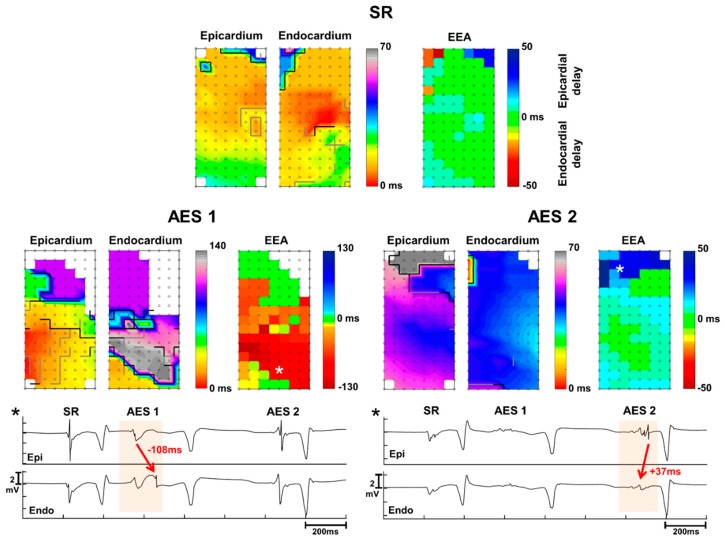
Presentation of severe endocardial delays during atrial extra systoles (AES). Delays reach up to 130 ms at the lower part and the upper part is refractory. However, with the second immediately following AES the delay shifts to the epicardium (epi) with delays up to 50 ms at the upper part. Electrogram examples demonstrate the severe delays at the endocardium (endo) for the first AES and at the epicardium for the second AES. Asterisks refer to the site of the electrograms in the corresponding maps. SR = sinus rhythm.

There was no clear preference for either epicardium or endocardium to lag during AES. However in AES with the most increase of EEA (>20% of the array), the endocardial side was delayed relative to the epicardium (Figure 4). The same side was often delayed in case of EEA during multiple AES within one patient. One of the patients demonstrated a change from endocardial to epicardial delay in two consecutive AES (Figure 5). The first AES of this patient also had the longest increase in EEA of all AES of all patients; the endocardium was delayed by up to 130 ms compared to the epicardium during AES. There was no effect of a history of atrial fibrillation on the degree of EEA during sinus rhythm or AES.

### 3.4. Impact of Prematurity and Aberrancy

There was no correlation observed between prematurity and EEA increase. AES with the largest increases in EEA had severe or complex aberrancy (Supplemental Figure S2). Median ΔEEA for AES with the mildest aberrancy was 0 (0–0)%, for AES with severe and complex aberrancy 0 (0–2.8)% and for AES with complex sinus rhythm activation pattern 0.7 (−1.9–+3.6)%. No difference was observed for ΔEEA in AES between aberrancy categories.

## 4. Discussion

### 4.1. Main Findings

The main finding of this study is that spontaneous AES demonstrate more EEA than sinus-beats. Maximal increase in EEA-delay observed with AES was 130 ms and maximal EEA-area increase was 47%. A possible cause for increased EEA during AES was an increase in the imbalance of epicardial versus endocardial conduction disorders. Both conduction delay and block increased during AES; conduction delay increased mainly at the epicardium whereas conduction block likely increases mainly at the endocardium.

### 4.2. Conduction Disorders in AES

The increase of high-resolution conduction disorders in AES especially for aberrant AES was previously reported [4]. The current study demonstrated that these conduction disorders also become more unequally distributed between the epicardium and endocardium in AES which may be one of the causes of increased EEA in AES. If conduction is delayed unevenly at epicardial and endocardial layers, this creates or enhances a substrate for asynchronous endo-epicardial activation. Studies have shown that conduction in AES mainly slows in the transverse direction, perpendicular to the myocardial fibers, thereby increasing the anisotropic behavior of the tissue [8,9,10]. This was underlined by our previous finding that aberrancy had an important role in aggravating conduction disorders during AES [4] The severity of transverse conduction disorders in diseased myocardium was related to the fibrosis architecture. Myocardium with clustered fibrotic areas demonstrated more AES induced conduction disorders than those with diffuse fibrosis [8,9]. Distribution between sub-epicardial and sub-endocardial atrial fibrosis has not been described so far, however is possibly very heterogeneously in nature. Nonetheless, the right atrial anatomy may favor endocardial conduction delays due to naturally limited transverse connections of the pectinate bundles making them vulnerable for conduction disorder in case of transversely travelling waves. In this study there was no clear dominance of endocardial delay. Although, the AES with the largest increases in areas of EEA did have endocardial delay. 

### 4.3. Arrhythmogenic Potential of AES and Effect of EEA

AES interrupt the stable rhythm originating from the sinus node by exciting the atria prematurely and/or from a site distant to the sinus node. As a result, at the time of the AES, recovery of myocardial cells can still be heterogeneously distributed in case of a premature AES. In the case of a different activation site during AES, the direction of activation changes leading to different conduction velocities due to anisotropy. Both of these concepts are able to result in re-entry in which an electrical pathway sustains itself, like a dog chasing its tail, causing a tachyarrhythmia [11,12]. The two prerequisites for re-entry are (1) unidirectional block and (2) a cycle length longer than the longest refractory (recovery) period. When unidirectional block occurs from the epicardium to the endocardium or vice versa, this will, in the best case, result in EEA only or it could result in an intramural re-entry circuit. This study demonstrated that either uni- or bidirectional conduction block between the layers occurs only during AES in patients.

Schuessler et al. have investigated endo-epicardial conduction at the right atrial appendage of dogs during sinus rhythm, pacing, extra-stimuli and induced tachyarrhythmia [13]. EEA increased with premature stimulation close to the refractory period and increased most with aberrant premature stimulation. Maximal recorded difference in excitation between epi- and endocardium during premature stimulation was 30 ms. They then induced tachyarrhythmia in the presence of high-dose acetylcholine with a single extra-stimulus and demonstrated an intramural re-entry pattern during tachyarrhythmia with a very short cycle length of approximately 60 ms and EEA up to 31 ms. In our study, we recorded a much larger endo-epicardial excitation difference of 130 ms in a spontaneous AES in a patient with aortic valve disease. Functional right atrial refractoriness has been measured at around 270 ± 30 ms during electrophysiological studies in patients with or without a history of atrial fibrillation at cycle lengths of 600 ms [14]. In human right atrial bundles perfused with pinacidil, intramural re-entry patterns with cycle lengths of 57 to 210 ms and maximal EEA of about half the re-entry cycle length (22–105 ms) were observed [15]. During atrial fibrillation, EEA >50 ms was demonstrated at the right atrium in patients with atrial fibrillation cycle lengths between 140–246 ms and at the left atrium in a goat-model of atrial fibrillation with cycle lengths around 120–140 ms [5,16]. In both studies, the majority of the frequent focal waves were most likely caused by transmural conduction as a result of EEA. These findings suggest that EEA of 130 ms could create an intramural pathway of long enough delay for atrial tissue to recover from refractoriness, thereby promoting re-entry. Besides re-entry around an unexcitable core, EEA could also result in spiral wave re-entry: if conduction between the layers is slowed down to an extent that the intramural wavefront curve starts to spiral.

### 4.4. Multifactorial Conditions for AES to Trigger Tachyarrhythmia

Multiple conditions can either contribute or counteract tachyarrhythmia initiation under different circumstances. One important factor is the refractory period, as it determines excitability of myocardial cells. Prematurity of AES close to the refractory period favors conduction disorders and inhomogeneous conduction due to uncompleted repolarization. However, the refractory period of myocardial cells is not a fixed time period and may vary under the influence of different factors. For example, the refractory period is rate-depended and also between atrial sites and within myocardial bundles differences in refractory duration exist [12]. Furthermore, conduction velocity depends on anatomical factors such as anisotropy and source-to-sink principles. Anisotropy is where an impulse travels slower along the transverse direction of myocardial bundles than the longitudinal direction. The source-to-sink principle is that conduction slows down when a small bundle has to excite a relatively large myocardial area [17]. The direction of the wavefront in the heterogeneous atrial architecture therefore determines conduction velocity. 

Next to the previously mentioned conditions for re-entry, a third one is required: the existence of a pathway for re-entry. If there are no connecting myocardial bundles between epicardium and endocardium at the site of EEA, there is no pathway for intramural re-entry. Finally, the atrial architecture can be disrupted in all three dimensions by underlying heart diseases or aging causing fibrosis or the conduction properties of myocardial cells change due to tachyarrhythmia induced electrical remodeling [8,9,18]. Atrial structural and electrical remodeling thereby influences conduction velocity and continuity. All these factors combined determine atrial sensitivity for an AES to initiate tachyarrhythmia. Although in our study the AES did not initiate tachyarrhythmia, the increase of epi-endocardial conduction disorders observed in AES add to arrhythmogenic tissue properties that underlie arrhythmia.

### 4.5. Clinical Impact

This study demonstrated that EEA aggravates during AES, which thereby increases the arrhythmogenic properties of AES. Importance of EEA therefore seems not confined to atrial fibrillation alone. EEA has also been suggested as a mechanism during atrial flutter [19]. Clinicians need to be aware that current mapping technologies in patients are limited to diagnosing conduction disturbances on only one side of the atrial wall. There is a whole black box of atrial activation on the other side, especially during arrhythmia and arrhythmia prone conditions such as AES.

### 4.6. Study Limitations

No atrial tachyarrhythmia was initiated by the AES in this study, as is the case for most AES, and most patients also did not have a history of atrial arrhythmias. Multiple factors contribute to tachyarrhythmia initiation. Due to the limited size of our study group and frequent occurrence of multiple AES in a short recording time period, this study could not substantially determine the effect of prematurity or aberrancy on increase of EEA during AES. Endo-epicardial mapping with performance of pacing protocols including premature stimulation could elucidate the contribution of AES characteristics on EEA occurrence. Furthermore, endo-epicardial mapping during cardiac surgery is mostly restricted to the right atrium, therefore left atrial data is limited. 

## 5. Conclusions

Atrial ectopic activation interrupting sinus rhythm provokes more conduction disorders between the epicardium and endocardium which creates or aggravates EEA. An imbalance in the degree of conduction disorders at the epicardium versus the endocardium could attribute to the occurrence of EEA in AES. Asynchrony between the layers in AES reached up to 130 ms which could be enough to initiate an intramural re-entry pathway under the right conditions. Current mapping tools in patients are not equipped to recognize arrhythmogenic properties between epi- and endocardium.

## Figures and Tables

**Table 1:** Conduction disorders during sinus rhythm vs atrial extrasystole.

	Sinus Rhythm		Atrial Extrasystole		*p*-Value
**Overall**					
CB (mm)	8	(0–48)	20	(6–41)	0.020
CD (mm)	24	(12.5–36)	38	(18.5–50)	0.014
Epicardium					
CB (mm)	2	(0–16)	4	(0–15.5)	0.639
CD (mm)	11	(4–19.5)	16	(8–27.5)	0.030
Endocardium					
CB (mm)	4	(0–26)	14	(5–23.5)	0.113
CD (mm)	12	(6–22)	16	(8–26)	0.276
Endo- vs. Epicardium					
∆CB (mm)	2	(0–8)	10	(2–17.5)	0.037
∆CD (mm)	6	(2–11.5)	7	(4–14)	0.102
Endo-Epicardial Asynchrony					
EEA ≥ 10 ms (%)	0	(0–2.3)	0.7	(0–6.0)	0.023
EEA ≥ 15 ms (%)	0	(0–0.6)	0	(0–3.0)	0.053

CB = conduction block, CD = conduction delay, EEA = endo-epicardial asynchrony.

## Supplementary Materials

The following are available online at https://www.mdpi.com/2077-0383/9/2/558/s1, Table S1: Clinical characteristics, Figure S1: Epicardial and endocardial conduction delay (CD) and block (CB) during SR and AES, Figure S2: EEA difference for AES compared to SR per aberrancy category.
