# Supplementary material for: Atrial Ectopy Increases Asynchronous Activation of the Endo- and Epicardium at the Right Atrium
Source: J Clin Med. 2020 Feb 18;9(2):558. doi: 10.3390/jcm9020558 (PMC7074567; doi:10.3390/jcm9020558)
Supplement: Supplementary file 1 [file jcm-09-00558-s001.pdf]

Supplementary materials

Supplemental Table S1. Clinical characteristics.

**Total**	***N* = 23**
Age, years	64 ± 9
Male/Female	18/5
Median number of AES (min–max)	2 (1–7)
Current Heart Disease, *N* (%)	
Coronary artery disease	17 (74)
Aortic valve disease	4 (17)
Mitral valve disease	7 (30)
Tricuspid valve disease	1 (4)
Patent foramen ovale	1 (4)
Left ventricular dysfunction	4 (17)*
Left atrial enlargement	7 (30)
Medical History, *N* (%)	
Hypertension	13 (57)
Hypercholesterolemia	10 (43)
Diabetes mellitus	8 (35)
Atrial Fibrillation	9 (39)
Paroxysmal	7 (30)
Persistent	2 (9)
Anti-arrhythmical medication, *N* (%)	
Class 1	0 (0)
Class 2	17 (74)
Class 3	1 (4)
Class 4	3 (13)
Digoxin	3 (13)

* 3 mild and 1 moderate dysfunction.

Supplemental Figure S1.

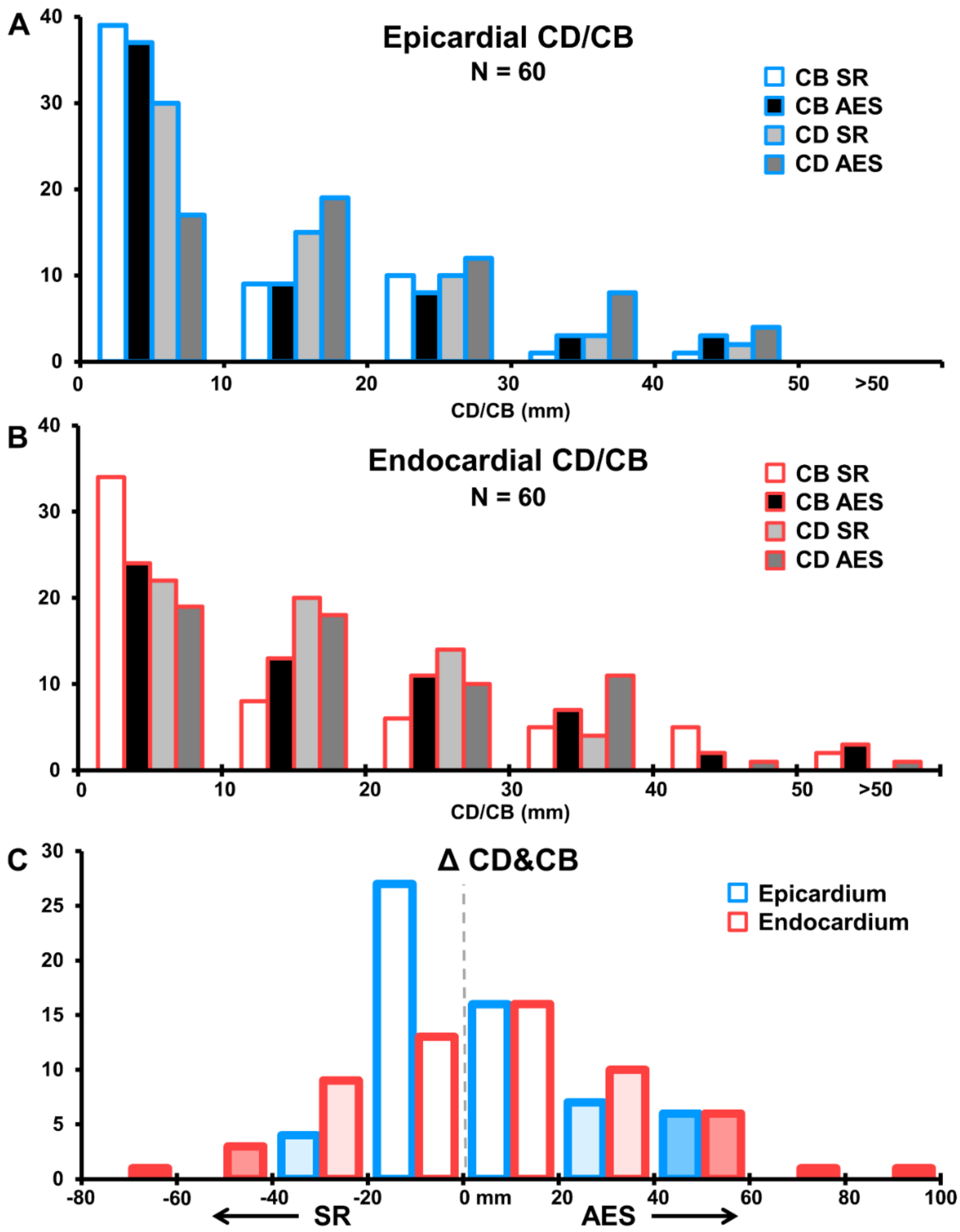

Figure 1. Epicardial and endocardial conduction delay (CD) and block (CB) during SR and AES. Frequency histograms of epicardial (A) and endocardial (B) CD and CB during SR and AES. Bin limits are set at increases of 10mm. C: frequency histogram of the difference of total CD and CB in AES compared to SR for epicardium and endocardium separately. Positive values represent an increase and negative values a decrease of conduction disorders. Bin limits are set at steps of 20mm.

Supplemental Figure S2

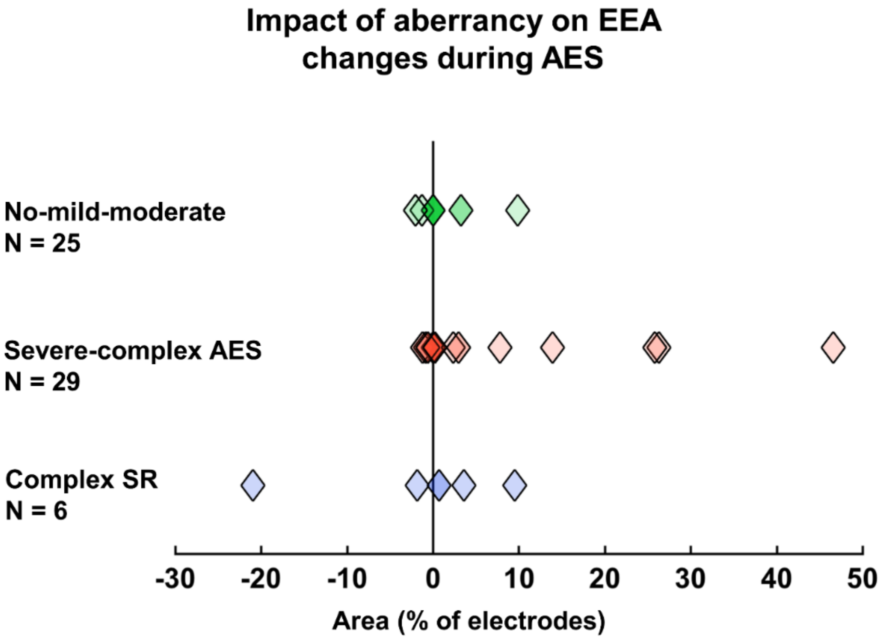

Figure 2. EEA difference for AES compared to SR per aberrancy category. Aberrancy categories include AES with no, mild or moderate aberrancy vs AES with severe or complex activation patterns during AES vs AES with complex activation patterns during SR only. Largest increases in EEA occurred in AES with severe/ complex activation patterns. A complex pattern during only SR decreased EEA by 21% in one patient.
